# Responses among substance abuse treatment providers to the opioid epidemic in the USA: Variations in buprenorphine and methadone treatment by geography, operational, and payment characteristics, 2007-16

**DOI:** 10.1371/journal.pone.0229787

**Published:** 2020-03-03

**Authors:** Justin C. Yang, Andres Roman-Urrestarazu, Carol Brayne

**Affiliations:** 1 Cambridge Institute of Public Health, University of Cambridge, Cambridge, United Kingdom; 2 Department of Epidemiology and Applied Clinical Research, Division of Psychiatry, Faculty of Brain Sciences, University College London, London, United Kingdom; University of Washington, UNITED STATES

## Abstract

**Objective:**

To identify the geographic, organisational, and payment correlates of buprenorphine and methadone treatment among substance abuse treatment (SAT) providers.

**Methods:**

Secondary analyses of the National Survey of Substance Abuse Treatment Services (NSSATS) from 2007–16 were conducted. We provide bivariate descriptive statistics regarding substance abuse treatment services which offered buprenorphine and methadone treatment from 2007–16. Using multiple logistic regression, we regressed geographic, organisational, and payment correlates on buprenorphine and methadone treatment.

**Results:**

Buprenorphine is increasingly offered at SAT facilities though uptake remains comparatively low outside of the northeast. SAT facilities run by tribal governments or Indian Health Service which offer buprenorphine remain low compared to privately operated SAT facilities (AOR = 0.528). The odds of offering buprenorphine among facilities offering free or no charge treatment (AOR = 0.838) or a sliding fee scale (AOR = 0.464) was lower. SAT facilities accepting Medicaid payments showed higher odds of offering methadone treatment (AOR = 2.035).

**Conclusions:**

Greater attention towards the disparities in provision of opioid agonist therapies is warranted, especially towards the reasons why uptake has been moderate among civilian providers. Additionally, the care needs of Native Americans facing opioid-related use disorders bears further scrutiny.

## Introduction

The ongoing and sustained opioid epidemic in the United States has manifested as one of the most urgent contemporary public health problems such that the United States Centers for Disease Control and Prevention (CDC) added the crisis to its list of the top five public health challenges in 2014 [1]. Opioid overdose deaths have increased at an alarming and accelerated rate; from 2015–16, for instance, deaths due to opioid overdose increased 27.7% [2]. Opioid overdose mortality and its associated economic burden have been among the major consequences of this epidemic; in 2014, for instance, 61% of drug overdose deaths involved opioids [3] while health care spending among individuals with opioid abuse averages eight times higher than individuals without opioid abuse [4].

The burden of the opioid epidemic, involving both the misuse of prescribed opioids and the use of illicit opioids, has not manifested uniformly across the United States. One study found that drug poisoning mortality and opioid consumption rates varied significantly by state and region with drug poisoning rates highest in the Southwest and lowest in the Midwest [5]. Moreover, sales of opioid analgesic, which are prone to abuse, have varied significantly by state as well, with Alaska showing a 13 times higher methadone distribution than Nebraska in 2002 [5]. Another study has found that prescription drug abuse of oxycodone has been unevenly concentrated in eastern and southeastern states with a pattern of migration from the northeast and Appalachia towards the southeast and west [6].

Despite the self-evident need for effective opioid misuse treatment, systemic barriers to treatment persist [7, 8], particularly with respect to the type and mode of treatment [9, 10]. Because of the strict Federal regulatory oversight of pharmacotherapies for the treatment of opioid misuse, the accessibility or lack thereof of treatments such as buprenorphine and methadone has come under heightened scrutiny by researchers and practitioners [11] despite the fact that the American Psychiatric Association (APA) has endorsed the use of pharmacotherapy combined with psychotherapy as best practice [12, 13] and the American Society of Addiction Medicine (ASAM) has established the use pharmacotherapy for the treatment of opioid use disorder as a practice guideline [14]. Indeed, some practitioners have called for increased access to buprenorphine in the outpatient setting [15]. In addition, as broader patterns of macroeconomy have experienced a downturn at the start of the millennium, substance abuse treatment providers have responded with respect to changes to patient mix by changes in provider operators and acceptance of private health insurance [16].

In this study, we make use of a routine, annual survey of all substance abuse treatment (SAT) facilities in the United States to characterise the ways in which pharmacotherapeutic approaches to managing opioid use disorders, specifically buprenorphine and methadone, have or have not varied during ten recent years of the opioid epidemic from 2007–16, based on geographic, operational, and payment characteristics. By doing so, we seek to describe ongoing trends in SAT facility operations and whether or not disparities exist based on geographic, operational, and payment characteristics with respect to buprenorphine and methadone treatment, particularly given the clinical evidence supporting their use in opioid abuse treatment [17]. Finally, we provide some recommendations for policymakers to address issues facing the treatment of opioid use disorders based on our findings.

## Methods

Our study used data from the 2007–16 waves of the National Survey of Substance Abuse Treatment Services (N-SSATS), an annually repeated, survey of all known drug and alcohol abuse treatment facilities, both public and private, in the United States [18–27]. Given that the N-SSATS is a point-prevalence survey, where data is collected over a short, defined period of time in contrast to a longitudinal study, facilities are surveyed year after year [18–27]. The number of responses vary by year, with a minimum of 13,066 and a maximum of 14,162. The average number of responses per year was 13,614. In our analysis, we make use of 136,143 responses from 2007–16. The N-SSATS collects data regarding the location and characteristics of each substance abuse treatment (SAT) facility as well as the types of services offered [18–27]. Data is collected through mailed questionnaires, telephone interviews, and web-based surveys [18–27]. No adjustment is made for the approximately 5–10% facility nonresponse [18–27].

N-SSATS respondent location was categorized using Census Bureau Divisions (“division”) [28]: New England; Mid-Atlantic; East North Central; West North Central; South Atlantic; East South Central; West South Central; Mountain; and Pacific. SAT facility operating entities were defined as one of: private for-profit; private non-profit; state government; local government; tribal government; Department of Veterans Affairs (VA); Department of Defense (DoD); Indian Health Service (IHS); or other Federal agency [18–27]. SAT facility payment characteristics were also obtained from the N-SSATS. Dichotomous variables were coded to indicate whether a SAT facility provided no charge or free treatment, offered a sliding fee scale to patients, accepted cash payments, accepted private insurance, accepted Medicare, accepted Medicaid, accepted state (non-Medicaid) insurance, and accepted federal Military insurance [18–27].

Our outcome variables of interest were dichotomous variables coded to indicate whether a SAT facility offered specific pharmacotherapies: buprenorphine (with or without naltrexone) and methadone [18–27]. For our analyses of SAT facilities offering methadone, only facilities indicating the presence of an opioid treatment program (OTP) were assessed, as methadone dispensing is strictly regulated by Federal regulations and limited to OTPs [29].

All statistical analyses were performed in Stata 14 [30]. Bivariate descriptive analyses were conducted for our outcome variables of interest regarding service types and pharmacotherapies compared to facility characteristics, including geography, operating entity, and payment characteristics as detailed above. Multiple maximum-likelihood logit regressions weighted least squares were performed on these outcome variables, with and without adjustment, to yield odds ratios associated with SAT facility characteristics, specifically, geography, operating entity, and payment characteristics. The baseline characteristics used for logistic regressions were: New England for division, private for-profit for operating entity, and the counterfactual condition for payment characteristics (e.g. the counterfactual for “offers free or no charge treatment” would be “does not offer free or no charge treatment”). In addition to adjustment for our covariates of interest, all regression models also adjusted for year.

## Results

Descriptive characteristics for our study population are shown in Table 1.

**Table 1:** Characteristics of study population, 2007–16.

Year	2007	2008	2009	2010	2011	2012	2013	2014	2015	2016	χ^2^
n	13352	13404	13233	13066	13462	14056	13873	13914	13621	14162	
**Census Division**											*p*<0.001
New England	6.4%	6.5%	6.3%	6.3%	6.5%	6.7%	6.5%	6.6%	6.5%	6.8%	
Mid-Atlantic	13.4%	13.7%	13.8%	14.2%	13.9%	13.2%	13.0%	13.2%	13.2%	12.8%	
East North Central	15.8%	15.5%	15.3%	15.0%	15.1%	14.9%	15.1%	15.1%	14.9%	14.8%	
West North Central	8.4%	8.3%	8.2%	8.6%	8.8%	8.8%	8.6%	8.9%	9.0%	9.0%	
South Atlantic	15.6%	16.0%	16.0%	15.5%	15.8%	16.0%	16.4%	16.7%	17.0%	17.2%	
East South Central	5.6%	5.4%	5.4%	5.6%	5.7%	5.9%	5.8%	5.7%	5.8%	5.8%	
West South Central	6.8%	6.6%	6.6%	6.6%	6.5%	6.7%	6.8%	6.7%	6.5%	6.8%	
Mountain	8.6%	8.9%	8.8%	9.3%	9.2%	9.9%	10.1%	10.0%	10.3%	10.4%	
Pacific	19.4%	19.1%	19.6%	18.9%	18.6%	17.9%	17.6%	17.1%	16.8%	16.4%	
**Operating Entity**											*p*<0.001
Private-for-Profit	28.5%	29.3%	29.3%	30.0%	30.6%	31.1%	32.3%	33.0%	33.6%	35.4%	
Private Non-Profit	58.0%	58.0%	57.9%	57.5%	57.3%	56.3%	55.3%	55.2%	54.6%	53.2%	
State Government	3.1%	3.0%	2.9%	2.7%	2.4%	2.4%	2.3%	2.3%	2.4%	2.2%	
Local, County, or Community Government	6.6%	6.1%	6.0%	5.7%	5.5%	5.4%	5.3%	5.1%	5.1%	5.0%	
Tribal Government	1.4%	1.3%	1.4%	1.4%	1.6%	2.3%	2.1%	2.0%	1.9%	1.8%	
Department of Veterans Affairs	1.4%	1.3%	1.6%	1.7%	1.7%	1.5%	1.6%	1.5%	1.5%	1.5%	
Department of Defense	0.7%	0.7%	0.7%	0.7%	0.7%	0.6%	0.6%	0.6%	0.6%	0.6%	
Indian Health Service	0.3%	0.3%	0.3%	0.3%	0.2%	0.4%	0.3%	0.2%	0.2%	0.2%	
Other Federal Government Agency	0.1%	<0.05%	<0.05%	<0.05%	<0.05%	<0.05%	0.1%	0.1%	0.1%	<0.05%	
**Facility Characteristic**											
Operate an Opioid Treatment Program	8.3%	99.7%	99.7%	8.8%	8.8%	8.2%	9.2%	9.4%	9.8%	9.2%	*p*<0.001
**Types of Payment Assistance Offered**											
Offers no charge or free treatment	52.6%	52.1%	51.5%	49.6%	49.3%	49.5%	48.3%	47.1%	46.6%	45.5%	*p*<0.001
Sliding fee scale	62.8%	62.9%	62.8%	62.6%	62.7%	62.2%	62.4%	61.4%	60.6%	59.6%	*p*<0.001
**Accepted Payment Types**											
Cash or Self-Payment	91.3%	90.1%	91.1%	91.5%	91.6%	90.7%	91.3%	90.8%	90.5%	90.4%	*p*<0.001
Private Health Insurance	66.1%	64.7%	64.8%	65.8%	66.2%	66.8%	67.3%	68.7%	69.4%	70.5%	*p*<0.001
Medicare	35.8%	35.9%	34.2%	33.9%	34.1%	34.6%	34.6%	34.6%	35.1%	35.6%	*p =* 0.001
Medicaid	56.0%	55.5%	56.0%	57.0%	58.8%	60.3%	61.0%	62.1%	63.6%	63.8%	*p*<0.001
State-Financed Health Insurance	37.5%	41.3%	41.1%	42.0%	42.0%	43.4%	44.4%	47.4%	48.4%	49.2%	*p*<0.001
Federal Military Insurance	35.7%	35.4%	35.6%	35.5%	36.0%	36.5%	35.9%	36.7%	36.1%	36.5%	*p* = 0.36
**Pharmacotherapies Offered**											
Buprenorphine (with or without naloxone)	14.4%	15.2%	17.2%	18.6%	19.9%	20.7%	22.2%	23.4%	24.8%	27.2%	*p*<0.001
Methadone	11.2%	8.4%	11.4%	11.5%	11.3%	11.3%	11.7%	11.8%	12.1%	12.5%	*p*<0.001

**Fig 1:**
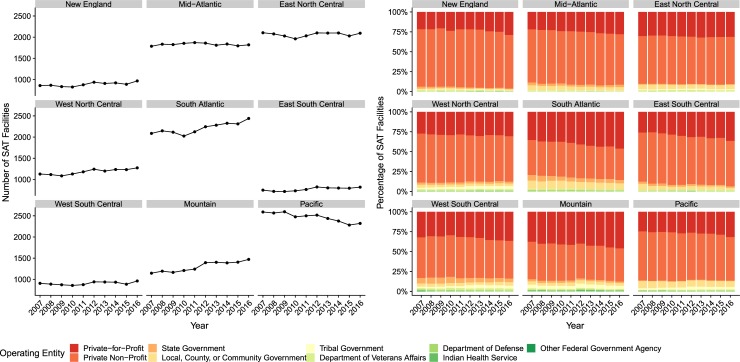
Number of SAT Facilities by Census Bureau Division, 2007–16, and Percentage of SAT Facilities by Operating Entity by Census Bureau Division, 2007–16.

As shown in Fig 1, the number of SAT facilities generally increased from 2007–16 across divisions except for Pacific where a decline in the number of SAT facilities from 2008 is observed. SAT facilities across all regions were mostly operated by private entities from 2007–16, ranging from 86.5% of all surveyed SAT facilities in 2007 to 88.6% in 2016.

**Fig 2:**
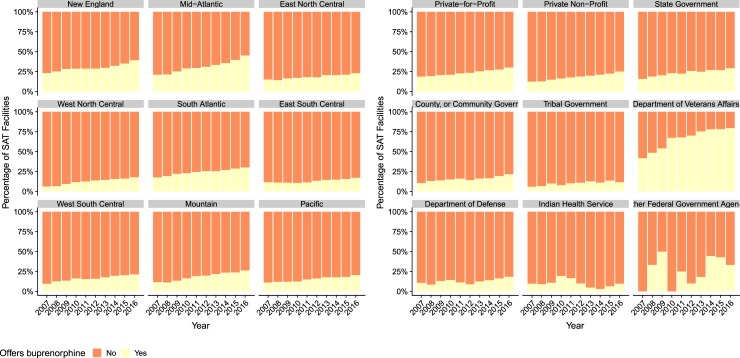
Percentage of SAT Facilities Offering Buprenorphine by Census Bureau Division, 2007–16, and Percentage of SAT Facilities Offering Buprenorphine by Operating Entity, 2007–16.

The percentage of SAT facilities offering buprenorphine increased from 2007–16 (χ^2^, nearly doubling from 14.4% of all SAT facilities to 27.2%, across all divisions as shown in Fig 2; nevertheless, less than half of SAT facilities in New England and Mid-Atlantic, and South Atlantic offered buprenorphine in 2016 and less than 25% did so in other divisions. Also shown in Fig 2, the percentage of SAT facilities buprenorphine increased from 2007–16 among SAT facilities whose operating entities were private-for-profit, private non-profit, state government, and VA, markedly so in the latter case where buprenorphine was offered at approximately three-quarters of SAT facilities operated by the VA in 2016. By contrast, less than 25% of SAT facilities operated by tribal governments, DoD or IHS offered buprenorphine from 2007–16.

**Fig 3:**
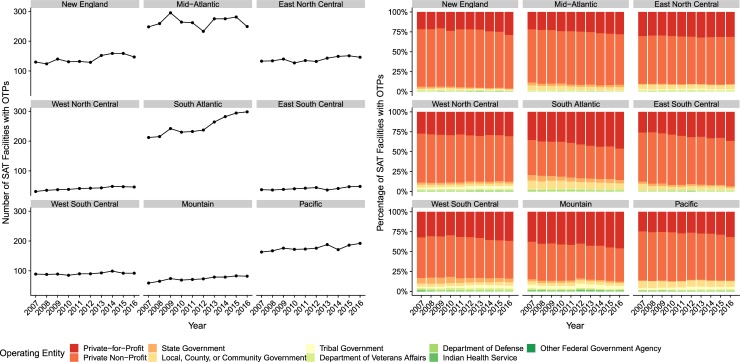
Number of SAT Facilities with OTPs by Census Bureau Division, 2007–16, and Percentage of SAT Facilities with OTPs Offering Methadone by Census Bureau Division, 2007–16.

Overall, the number of SAT facilities which operated an OTP remained stable from 2007–16, just below 10%. The number of SAT facilities which ran an OTP increased in South Atlantic and remained relatively constant in most other divisions as shown in Fig 3. In Mid-Atlantic, we observed an increase in the number of SAT facilities running an OTP in 2008, then declining until 2012 before increase again through to 2015. Reflecting broader patterns in facility operation noted above, SAT facilities running an OTP were predominantly operated by private entities.

**Fig 4:**
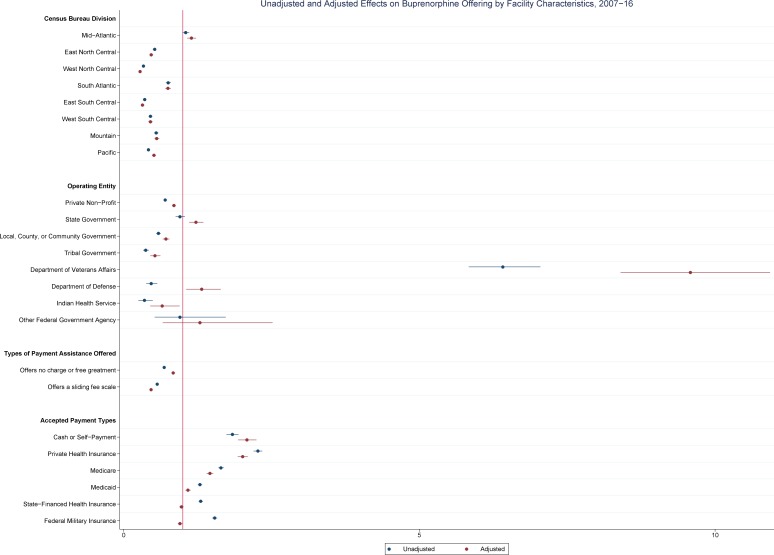
Unadjusted and adjusted effects on buprenorphine offering by SAT facility characteristics, 2007–16.

**Fig 5:**
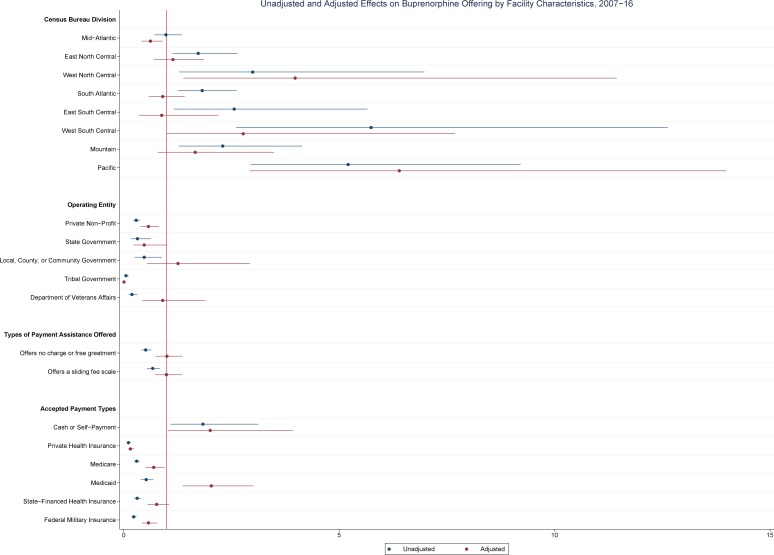
Unadjusted and adjusted effects on methadone offering by SAT facility characteristics (among SAT facilities reporting an OTP), 2007–16.

Table 2 shows the results of unadjusted and adjusted results of logistic regression of geographic, operational, and payment characteristics associated with whether SAT facilities offered buprenorphine and methadone pharmacotherapies from 2007–16. These results are also shown graphically in Figs 4 and 5. SAT facilities in most divisions, except for Mid-Atlantic (AOR: 1.15; 95% CI: 1.08–1.22), showed lower odds of offering buprenorphine treatment when compared to New England. SAT facilities operated by private non-profit entities (AOR: 0.85; 95% CI: 0.82–0.88), local, county, or community governments (AOR: 0.71; 95% CI: 0.66–0.77), tribal governments (AOR: 0.53; 95% CI: 0.45–0.62), or IHS (AOR: 0.65; 95% CI: 0.45–0.95) showed lower odds of offering buprenorphine while those operated by state government (AOR: 1.22; 95% CI: 1.11–1.35) or VA (AOR: 9.58; 95% CI: 8.40–10.93) showed higher odds of offering buprenorphine. SAT facilities offering any type of payment assistance, either charity care or a sliding fee scale, showed lower odds of offering buprenorphine than those that did not offer any form of payment assistance. SAT facilities accepting cash or self-payment (AOR: 2.09; 95% CI: 1.94–2.25), private health insurance (AOR: 2.01; 95% CI: 1.93–2.25), Medicare (AOR: 1.46; 95% CI: 1.40–1.52), or Medicaid (AOR: 1.09; 95% CI: 1.05–1.13) all showed higher odds of offering buprenorphine than those not accepting.

**Table 2:** Unadjusted and adjusted multiple logistic regression of SAT facility characteristics associated with pharmacotherapies offered.

	Buprenorphine				Methadone*			
	Unadjusted Odds Raios		Adjusted Odds Raios ***		Unadjusted Odds Raios		Adjusted Odds Raios ***	
**Census Bureau Division**								
New England	1		1		1		1	
Mid-Atlantic	1.051	(0.994–1.110)	1.147	(1.076–1.222)	0.981	(0.713–1.350)	0.621	(0.424–0.911)
East North Central	0.524	(0.494–0.555)	0.466	(0.437–0.498)	1.730	(1.132–2.642)	1.144	(0.702–1.864)
West North Central	0.336	(0.313–0.361)	0.278	(0.256–0.301)	2.993	(1.284–6.979)	3.979	(1.383–11.44)
South Atlantic	0.755	(0.715–0.797)	0.750	(0.704–0.799)	1.821	(1.266–2.621)	0.908	(0.583–1.415)
East South Central	0.354	(0.327–0.384)	0.320	(0.292–0.350)	2.566	(1.164–5.657)	0.882	(0.354–2.199)
West South Central	0.453	(0.421–0.486)	0.452	(0.416–0.491)	5.744	(2.614–12.62)	2.772	(0.999–7.692)
Mountain	0.551	(0.518–0.587)	0.559	(0.521–0.601)	2.298	(1.275–4.140)	1.661	(0.791–3.488)
Pacific	0.418	(0.395–0.442)	0.512	(0.479–0.548)	5.209	(2.945–9.214)	6.400	(2.929–13.98)
**Operating Entity**								
Private-for-Profit	1		1		1		1	
Private Non-Profit	0.704	(0.684–0.725)	0.849	(0.818–0.881)	0.292	(0.225–0.380)	0.574	(0.398–0.826)
State Government	0.954	(0.879–1.035)	1.223	(1.110–1.348)	0.322	(0.165–0.628)	0.475	(0.224–1.008)
Local, County, or Community Government	0.588	(0.550–0.628)	0.714	(0.660–0.772)	0.480	(0.260–0.885)	1.260	(0.542–2.928)
Tribal Government	0.374	(0.327–0.427)	0.528	(0.449–0.622)	0.0549	(0.0258–0.117)	0.00860	(0.00301–0.0246)
Department of Veterans Affairs	6.412	(5.836–7.045)	9.583	(8.403–10.93)	0.195	(0.121–0.313)	0.905	(0.432–1.893)
Department of Defense	0.466	(0.382–0.569)	1.319	(1.059–1.642)	**		**	
Indian Health Service	0.350	(0.249–0.492)	0.653	(0.450–0.947)	**		**	
Other Federal Government Agency	0.950	(0.523–1.726)	1.290	(0.661–2.519)	**		**	
**Types of Payment Assistance Offered**								
Offers no charge or free Tx	0.684	(0.666–0.702)	0.838	(0.809–0.867)	0.514	(0.411–0.643)	1.004	(0.740–1.360)
Uses sliding fee scale	0.568	(0.553–0.583)	0.464	(0.448–0.480)	0.674	(0.538–0.844)	0.994	(0.731–1.351)
**Accepted Payment Types**								
Accepts cash or self-payment	1.838	(1.740–1.942)	2.085	(1.936–2.245)	1.838	(1.081–3.125)	2.009	(1.027–3.931)
Accepts private health insurance	2.269	(2.197–2.343)	2.012	(1.930–2.097)	0.114	(0.0791–0.165)	0.157	(0.101–0.245)
Accepts Medicare payments	1.646	(1.602–1.691)	1.458	(1.402–1.515)	0.304	(0.242–0.381)	0.696	(0.510–0.950)
Accepts Medicaid payments	1.291	(1.256–1.327)	1.088	(1.045–1.132)	0.521	(0.396–0.686)	2.035	(1.372–3.017)
Accepts state-financed health insurance	1.303	(1.268–1.339)	0.977	(0.941–1.015)	0.313	(0.246–0.398)	0.767	(0.557–1.055)
Accepts Federal military insurance	1.540	(1.497–1.583)	0.953	(0.917–0.990)	0.232	(0.183–0.294)	0.575	(0.426–0.776)

*Logistic regression was run only for facilities indicating a registered OTP

**No facilities operated by DoD, IHS, another Federal government agency indicated they registered OTP

*** Adjusted for year, geography, operating entity, and payment characteristics

For methadone treatment, different trends among geographic, operational, and payment characteristics were found among SAT facilities operating an OTP. Compared to SAT facilities in New England, SAT facilities in Pacific showed higher odds of offering methadone treatment (AOR: 6.40; 95% CI: 2.93–13.98). Of the SAT facilities which responded to the N-SSATS from 2007–16, no SAT facilities operated by DoD ran an OTP and no SAT facilities operated by IHS or another Federal government agency offered methadone treatment. SAT facilities operated by a private non-profit entity (AOR: 0.57; 95% CI: 0.40–0.83) or by tribal government (AOR: <0.01) showed lower odds of offering methadone treatment than SAT facilities operated by a private-for-profit entity, particularly so in the latter case. SAT facilities accepting private health insurance (AOR: 0.16; 95% CI: 0.10–0.25) and Medicare (AOR: 0.70; 95% CI: 0.51–0.95) showed lower odds of offering methadone treatment than those that did not accept those forms of payment while SAT facilities accepting Medicaid showed double the odds of offering methadone treatment than SAT facilities not accepting Medicaid (AOR: 2.04; 95% CI: 1.37–3.02).

## Discussion

Our results showed changes in the SAT facility landscape from 2007–16 and highlighted differences in buprenorphine and methadone treatment availability based upon geographic, operational, and payment characteristics. Firstly, though it is promising to see that buprenorphine is increasingly offered at SAT facilities among all divisions, uptake remained low outside of the northeast; we observe that all regions, except for Mid-Atlantic, showed lower odds of SAT facilities offering buprenorphine treatment. Moreover, though SAT facilities across a range of operational entities increasingly offered buprenorphine treatment, the percentage of SAT facilities run by tribal governments or IHS which offered buprenorphine remains low, highlighting a potential disparity in treating Native Americans. Though SAT facilities operated by the VA which offer buprenorphine have rapidly increased, growth has been more attenuated among facilities run by other operational entities as shown by the odds of SAT facilities offering buprenorphine among these SAT facilities. The odds of offering buprenorphine among facilities offering payment assistance was lower than those without payment assistance. For methadone, on the other hand, we observed that SAT facilities accepting Medicaid payments showed higher odds of offering methadone treatment.

Our analyses are limited by the shortcomings of data drawn from the N-SSATS. Given major changes to survey design in 2007, namely the exclusion of questions capturing our independent and dependent variables of interest, inclusion of prior waves of the N-SSATS which could have extended our study period was not possible. Moreover, the N-SSATS does not collect information regarding client mix among SAT facilities. As client mix and needs contribute to the determination of individual SAT facility service offerings, this omission may have contributed to omitted variables bias. Nevertheless, given that the N-SSATS is intended to be a comprehensive annual survey of all SAT facilities in the United States, our analysis is strengthened as we can analyse nearly all SAT facilities rather than relying on a generalizable sample [18–27]. Our use of a ten-year long study period further contributes to our attempts to mitigate bias. In addition, to our knowledge, no other dataset currently exists which documents SAT facilities on a national level to the extent that the N-SSATS provides.

Given the magnitude of the opioid epidemic and its sustained impact on population health, our findings suggest that greater attention towards the provision of pharmacotherapies for the treatment of opioid-related use disorders is warranted, such as understanding the reasons why uptake of pharmacotherapies for the treatment of opioid-related use disorders has been moderate among civilian healthcare providers. This can complement ongoing and sustained efforts to alter prescribing practices [31] and to generate novel formulations of abuse-deterrent opioid analgesics [32]. The relatively low numbers of civilian SAT facilities reporting the availability of buprenorphine therapy is concerning, given research which suggests that drug-free treatments may contribute towards greater patient mortality than medication-assisted therapy [33]. Though buprenorphine prescription has, to date, been strictly limited by law, researchers have called on lawmakers to consider relaxing such restrictions as they may contribute to greater patient harm [34]. Previous research has found a similar trend regarding the supply of waivered physicians in the northeast relative to other regions [35] and though some have offered explanations for this with respect to health care reform [36] or regional variations in opioid mortality [37], more research is needed to better understand the factors which have led to the relatively low uptake of buprenorphine treatment outside of the northeast. A number of general causes have been suggested as contributory towards the relatively low use of buprenorphine treatment, including a shortage of certified prescribers and worries about patient diversion [38, 39] as well as insufficient prescriber knowledge about the use of opioid agonist therapy and potential stigma associated with its use [40].

Our findings are consistent with other analyses of pharmacotherapy among providers primarily focused on Native Americans which have also identified low rates of pharmacotherapy implementation for substance abuse treatment [41]. In the case of opioids specifically, some qualitative research suggests that the high prevalence of oxycodone use among a tribal community has led to heightened sensitivity regarding opioid misuse [42], suggesting that approaches to increasing the use of pharmacotherapy for opioid-related substance use disorders must be culturally appropriate and contextual to the needs of Native Americans [43]. Developing cultural competence and capacity for evidence-based treatment of opioid-related substance disorders will contribute greatly to addressing the disparities we have identified.

With respect to methadone treatment, the relatively static number of SAT facilities with OTPs over time may be cause for concern as the number of individuals with opioid-related use disorders continues to increase. One study assessing the gap between treatment need and capacity for OTPs found that 96% of states showed opioid abuse or dependence rates higher than their OTP capacity rates with 38 states reporting at least 75% of their OTPs were operating at 80% capacity or more [11]. One way of addressing the lack of growth of OTPs is by devoting greater resources towards expanding the number of waivered physicians who can provide buprenorphine in the physician office setting, particularly among rural areas which have been underserved with respect to opioid-related substance disorder treatment [44]. Another approach is to relax the patient limits set on waivered physicians which can be a less resource-intensive method of increasing capacity for care without the need to increasing the number of SAT facilities, OTP programs, or waivered physicians [45].

Opioid use disorder continues to present pressing and urgent challenges to public health. SAT facilities, which serve a crucial role in the treatment of opioid use disorder, have generally shown an increase in the use of buprenorphine yet this increase was not observed as strongly among facilities offering payment assistance, a characteristic which may determine patient access to treatment. Nevertheless, our observation that facilities accepting Medicaid payments showed higher odds of offering methadone treatment suggests that there may be other opportunities where low-income, uninsured, or underinsured patients may receive medicated-assistant treatment. Still, much more research is needed to understand how SAT facilities as a whole, whether by policy or in practice, have responded to the opioid crisis. More nuanced approaches to understanding local and regional contexts for opioid use disorders and their treatment will enable more responsive care planning and service provision where it is needed most, for instance, among areas with high populations of Native Americans.
